# Supplementary figures and images for: An Efficient GUI-Based Clustering Software for Simulation and Bayesian Cluster Analysis of Single-Molecule Localization Microscopy Data
Source: Front Bioinform. 2021 Oct 11;1:723915. doi: 10.3389/fbinf.2021.723915 (PMC9581037; doi:10.3389/fbinf.2021.723915)

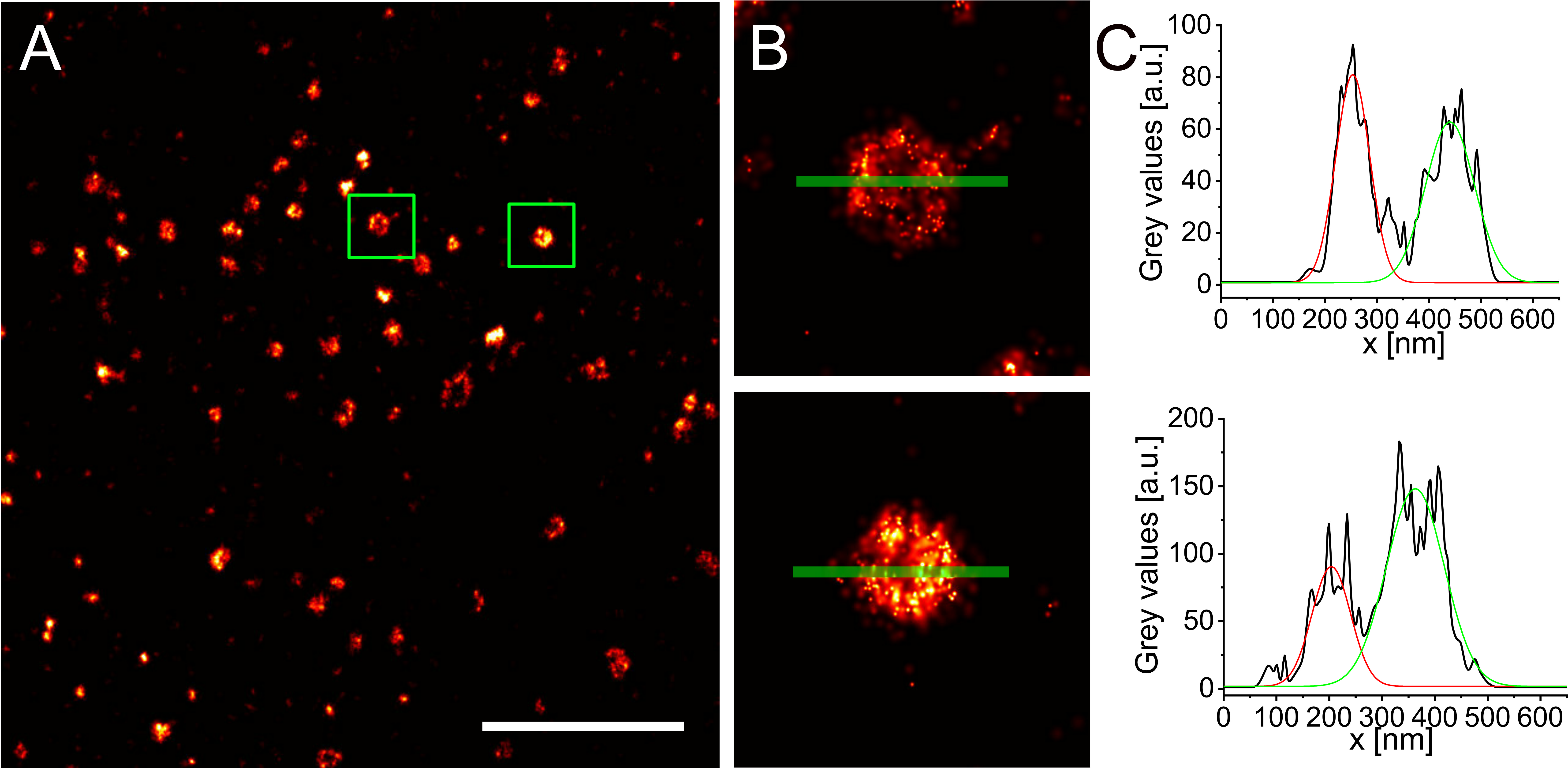

Supplement: Supplementary file 1 [file Image1.TIF]
