# Supplementary material for: An Efficient GUI-Based Clustering Software for Simulation and Bayesian Cluster Analysis of Single-Molecule Localization Microscopy Data
Source: Front Bioinform. 2021 Oct 11;1:723915. doi: 10.3389/fbinf.2021.723915 (PMC9581037; doi:10.3389/fbinf.2021.723915)
Supplement: Supplementary file 2 [file DataSheet1.PDF]

Supplementary Material

1 SUPPLEMENTARY FIGURES

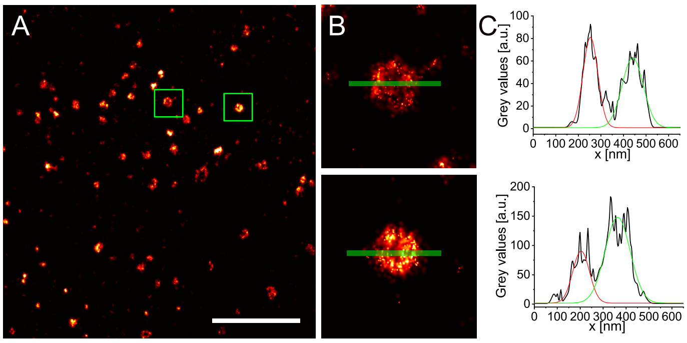

Figure S1. Clathrin-coated pit size (A) HeLa cells with stable expression of CLC-GFP were stained with anti-GFP nanobody labeled with Alexa Fluor 647, and (*d*)STORM images were taken. The scale bar is 3 µm. **(B)** Zoom-in on two individual clathrin-coated pits in *(A)* and line profiles were taken. The size of the image is 1 µm x 1 µm. **(C)** Fitting the line profiles with two Gaussians results in a diameter of 126 $\pm$ 24 nm (mean $\pm$ standard deviation, N = 9).
